# Comparative study of ^1^H-NMR metabolomic profile of canine synovial fluid in patients affected by four progressive stages of spontaneous osteoarthritis

**DOI:** 10.1038/s41598-024-54144-3

**Published:** 2024-02-13

**Authors:** Angela Palumbo Piccionello, Sara Sassaroli, Luca Pennasilico, Giacomo Rossi, Alessandro Di Cerbo, Valentina Riccio, Caterina Di Bella, Luca Laghi, Maddalena Angelini, Carlotta Marini, Gian Enrico Magi

**Affiliations:** 1 School of Biosciences and Veterinary Medicine, University of Camerino, 62024 Matelica, Italy; 2 Department of Agro-Food Science and Technology, University of Bologna, 47023 Cesena, Italy

**Keywords:** Cartilage, Metabolomics

## Abstract

The study aimed to assess the metabolomic profile of the synovial fluid (SF) of dogs affected by spontaneous osteoarthritis (OA) and compare any differences based on disease progression. Sixty client-owned dogs affected by spontaneous OA underwent clinical, radiographic, and cytologic evaluations to confirm the diagnosis. The affected joints were divided into four study groups based on the Kallgreen–Lawrence classification: OA1 (mild), OA2 (moderate), OA3 (severe), and OA4 (extremely severe/deforming). The osteoarthritic joint’s SF was subjected to cytologic examination and ^1^H-NMR analysis. The metabolomic profiles of the study groups’ SF samples were statistically compared using one-way ANOVA. Sixty osteoarthritic joints (45 stifles, 10 shoulders and 5 elbows) were included in the study. Fourteen, 28, and 18 joints were included in the OA1, OA2, and OA3 groups, respectively (0 joints in the OA4 group). Metabolomic analysis identified 48 metabolites, five of which were significantly different between study groups: Mannose and betaine were elevated in the OA1 group compared with the OA2 group, and the 2-hydroxyisobutyrate concentration decreased with OA progression; in contrast, isoleucine was less concentrated in mild vs. moderate OA, and lactate increased in severe OA. This study identified different ^1^H-NMR metabolomic profiles of canine SF in patients with progressive degrees of spontaneous OA, suggesting ^1^H-NMR metabolomic analysis as a potential alternative method for monitoring OA progression. In addition, the results suggest the therapeutic potentials of the metabolomic pathways that involve mannose, betaine, 2-hydroxyisobutyrate, isoleucine, and lactate.

## Introduction

Osteoarthritis (OA) is a common chronic disease that affects the entire joint^1^. It is characterized by inflammation, alteration of synovial fluid, progressive deterioration of articular cartilage, synovial membrane dysfunction, subchondral bone sclerosis, and formation of periarticular osteophytes^2^, with the depletion of matrix protein driven by proteases including multiple matrix metalloproteinases (MMPs) and a disintegrin and metalloproteinase with thrombospondin motifs (ADAMTSs)^3^. OA is responsible for significant pain, reduction of joint mobility until loss of joint function, and disability in humans and dogs^4^. OA is a multifactorial pathology, and age, sex, obesity, activity level, prior joint injury, and inherited susceptibility are recognized risk factors^5^. Also, immune system activity and metabolic disorders can influence the development and progression of OA^6^. The incidence of this pathology is high in canines and humans^10^, and, in particular, it was estimated that affects 20% of the canine population over 1 year of age^5^ excluding possible cases not reported in veterinary medicine.

Currently, OA can be detected in the later stages of the disease because it is diagnosed predominantly through clinical examination and diagnostic imaging^12^, and there is no definitive treatment that can stop the progression and cure the disease^11^. For this reason, the scientific literature has increased its interest in researching novel therapies^13^^,^^14^ and alternative detection methods for early diagnosis and monitoring of disease activity and progression^15^.

Recently, studies have focused on metabolomics to detect OA’s metabolic fingerprint and describe and identify related biomarkers. Metabolomics is the study of the most abundant small molecules (< 1500 Da) in a biological system to understand the metabolic changes occurring during physiological and pathological conditions^12^. Different studies have attempted to identify biochemical biomarkers in urine^19^, plasma, blood serum^21^, and cerebrospinal fluid^23^, but the most common biofluid used in OA research is synovial fluid (SF)^12^. SF is in contact with numerous tissues altered during joint pathology (synovial membrane, cartilage, and subchondral bone), and the degradation products, enzymes, and signal transduction molecules involved in OA are first released from the cartilage matrix into SF. SF should, therefore, yield the most detailed available profile of joint metabolism, and it holds significant potential in the discovery of biomarkers whose levels are altered in the early stages of disease progression^24^. In healthy and pathologic joints of humans and animals, the metabolomic SF profile was recently investigated by ^1H^H-nuclear magnetic resonance spectroscopy (H-NMR), an analytical technique widely used in metabolomics analysis thanks to its capacity to quantify rapidly and simultaneously, with the same sensitivity, a large number of metabolites, requiring minimal sample pretreatment^24^.

Studies on the metabolomics of the SF of domestic animals affected by spontaneous OA are poor, and their results are not always in agreement with each other^25^. They have suggested a significant change in the concentration of metabolites due to the OA condition, emphasizing the complex mechanism underlying OA and the potential diagnostic and prognostic capacity of the metabolomic analysis. For this purpose, no studies have investigated the metabolic changes in SF of domestic animals during the OA progression.

The current study aimed to assess the metabolomic profile of the SF of dogs affected by spontaneous OA and compare any differences in the four groups of progressive degrees of OA. The study’s results could provide new information about the metabolic shifts induced by the evolution of OA not only in the dogs, but also in the human species. Indeed, the client-owned dogs are excellent models of human disease, because they share the same living environments and food resources and because the diseases are similar in physiology, presentation, and therapy response^18^.

## Results

### Animals, joints enrolled and cytological analysis

Seventy-two client-owned dogs of different breeds showing unilateral lameness were referred by the Veterinary Teaching Hospital of the University of Camerino. Of these, five patients were excluded from the study because metabolic and neoplastic pathologies were detected during clinical evaluations, and seven patients were excluded because cytological examination of SF detected a suppurative or immunomediated arthropathy. Sixty dogs, 24 males and 36 females, respected the inclusion criteria (mean ± SD age, 8.25 ± 2.41 years, and mean ± SD weight, 21.33 ± 9.19 kg), and they were enrolled in the study. Sixty joints were analyzed in the study, specifically 45 stifles, 10 shoulders, and 5 elbows (Table 1).

**Table 1:** Breed, sex, bodyweight (BW), joints and pathology of patient enrolled in the study (osteoarthritis, OA; rupture of cranial crucial ligament, RCCL; osteochondritis dissecans, OCD; elbow dysplasia, ED).

Case	Breed	Sex	Age (year)	BW(kg)	Joint	Pathology	Group
1	Mix breed	F	11	19	Stifle	OA secondary to RCCL	1
2	Rottweiler	F	12	26	Stifle	OA secondary to RCCL	1
3	Corso	M	6	45	Shoulder	OA secondary to OCD	1
4	Mix breed	F	5.5	20	Stifle	OA secondary to RCCL	1
5	Labrador retriever	M	1	25	Stifle	OA secondary to RCCL	1
6	Mix breed	F	3	17	Stifle	OA secondary to RCCL	1
7	American St. terrier	F	2	34	Stifle	OA secondary to RCCL	1
8	Labrador retriever	F	4	31	Stifle	OA secondary to RCCL	1
9	Pitbull	M	1	26	Stifle	OA secondary to RCCL	1
10	English Pointer	M	3	23	Stifle	OA secondary to RCCL	1
11	Mix breed	M	9	18	Shoulder	OA	1
12	Alaskan Malamute	M	6.5	34	Stifle	OA secondary to RCCL	1
13	Mix breed	M	10	15	Stifle	OA secondary to RCCL	1
14	American Bully	F	1	27	Stifle	OA secondary to RCCL	1
15	German Shepherd	M	8.5	34	Stifle	OA secondary to RCCL	2
16	Mix breed	F	1.5	24	Stifle	OA secondary to RCCL	2
17	Rottweiler	F	12	26	Stifle	OA secondary to RCCL	2
18	Mix breed	F	11	21	Stifle	OA secondary to RCCL	2
19	Boxer	F	11	25	Stifle	OA secondary to RCCL	2
20	Labrador retriever	M	3.5	27	Stifle	OA secondary to RCCL	2
21	Maremma shepherd	F	10	37	Shoulder	OA	2
22	Mix breed	F	4	18	Shoulder	OA	2
23	Mix breed	F	10	37	Shoulder	OA	2
24	Alaskan Malamute	M	10	36	Shoulder	OA	2
25	Mix breed	F	6	16	Shoulder	OA	2
26	Mix breed	M	9	29	Shoulder	OA	2
27	Mix breed	F	6	24	Stifle	OA secondary to RCCL	2
28	Mix breed	F	4	17	Stifle	OA secondary to RCCL	2
29	Mix breed	F	6	19	Stifle	OA secondary to RCCL	2
30	Rottweiler	F	5	32	Stifle	OA secondary to RCCL	2
31	Labrador retriever	M	1	23	Elbow	OA secondary to ED	2
32	Braque Saint-Germain	F	6	19	Stifle	OA secondary to RCCL	2
33	American St. terrier	M	2	37	Stifle	OA secondary to RCCL	2
34	Mix breed	F	5	15	Stifle	OA secondary to RCCL	2
35	Mix breed	F	5	12	Stifle	OA secondary to RCCL	2
36	Mix breed	M	6	21	Stifle	OA secondary to RCCL	2
37	Corso	M	4	41	Stifle	OA secondary to RCCL	2
38	English Pointer	M	3	23	Shoulder	OA	2
39	Mix breed	F	9	16	Stifle	OA secondary to RCCL	2
40	Mix breed	M	7	22	Stifle	OA secondary to RCCL	2
41	Poodle	F	6.5	10	Stifle	OA secondary to RCCL	2
42	German Shepherd	M	7	24	Stifle	OA secondary to RCCL	2
43	Mix breed	M	2	10	Stifle	OA secondary to RCCL	2
44	German Shepherd	F	12	37	Stifle	OA secondary to RCCL	3
45	Rottweiler	F	7	32	Stifle	OA secondary to RCCL	3
46	Labrador retriever	M	2.5	28	Stifle	OA secondary to RCCL	3
47	Mix breed	F	2	23	Elbow	OA secondary to ED	3
48	Mix breed	M	2	17	Stifle	OA secondary to RCCL	3
49	Mix breed	F	6	19	Stifle	OA secondary to RCCL	3
50	Mix breed	M	1	36	Stifle	OA secondary to RCCL	3
51	Maremma shepherd	M	2	39	Stifle	OA secondary to RCCL	3
52	American St. terrier	F	7	28	Stifle	OA secondary to RCCL	3
53	Mix breed	F	12	22	Elbow	OA secondary to ED	3
54	Petit Blue de Gascogne	M	8	25	Stifle	OA secondary to RCCL	3
55	Mix breed	F	12	22	Stifle	OA secondary to RCCL	3
56	Labrador retriever	F	7.5	32	Stifle	OA secondary to RCCL	3
57	Rottweiler	F	12	26	Elbow	OA secondary to ED	3
58	Mix breed	F	2	23	Elbow	OA secondary to ED	3
59	Mix breed	F	12	22	Stifle	OA secondary to RCCL	3
60	Pitbull	M	8	25	Stifle	OA secondary to RCCL	3

The OA1 group included 14 joints (11 stifles, 3 shoulders), the OA2 group included 29 joints (21 stifles, 7 shoulders, 1 elbows), the OA3 included 17 joints (13 stifles and 4 elbows), and the OA4 group included 0 joints. Therefore, three study groups were considered (OA1, OA2, and OA3 groups). In the OA1 was included 7 males and 7 females, in the OA2 group 11 males and 18 females, and in the OA3 group 6 males and 11 females. In the OA1, OA2, and OA3 groups, the mean ± SD patients’ age was 5.36 ± 3.86, 6.24 ± 3.08, and 6.82 ± 4.14 years, respectively. Peso corporeo (BW) mean ± SD was 25.71 ± 8.20 kg, 24.31 ± 8.75 kg, and 26.82 ± 6.40 kg, respectively. There were no statistically significant differences in the ages and BW among the three groups.

Considering the number of stifle joints included in our study compared to other joints, a statistical comparison of the concentration of metabolites identified in the SF of the stifles, affected by rupture of cranial cruciate ligament, was performed. In particular, the OA1_stifle_ (n = 11 stifle joints), OA2_stifle_ (n = 21 stifle joints) and OA3_stifle_ (n = 13 stifle joints) groups were compared. In the OA1_stifle_, OA2_stifle_, and OA3_stifle_ groups, the mean ± SD patients’ age was 5.18 ± 4.17, 6.09 ± 2.94, and 6.76 ± 3.82 years, respectively. BW mean ± SD was 24.91 ± 6.55 kg, 23.14 ± 8.73 kg, and 27.85 ± 7.01 kg, respectively. There were no statistically significant differences in the ages and BW among the three groups.

**Figure 1:**
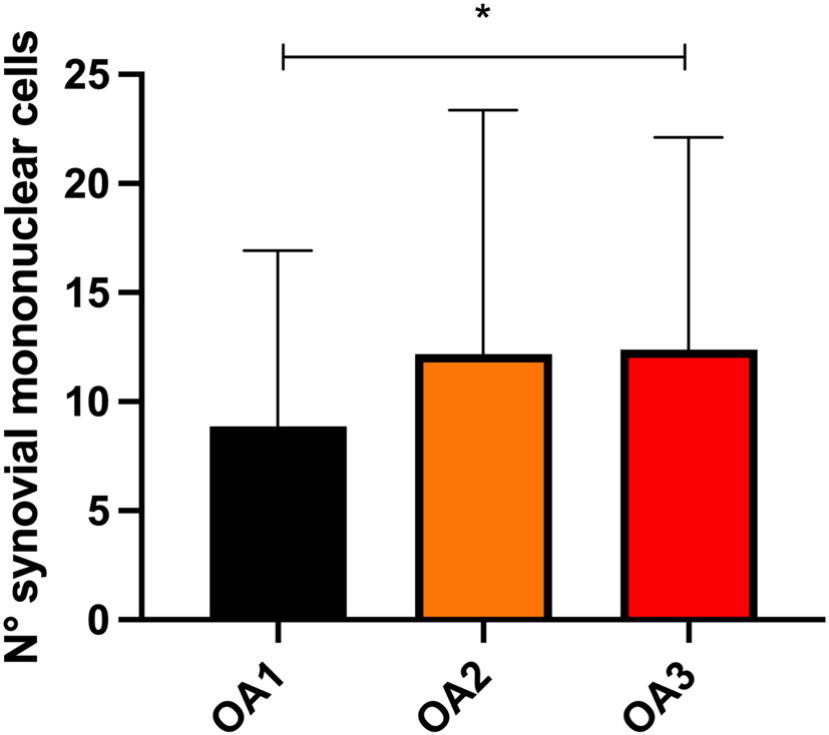
Comparison of the number of inflammatory cells in OA1, OA2 and OA3 groups. Asterisk (*) indicates a significant difference (*p* < 0.05) between groups.

At semi-quantitative cytological analysis, the mean ± SD of the number of inflammatory cells in OA1, OA2 and OA3 groups was of 8.8 ± 5.71, 12.23 ± 9.05 and 12.38 ± 8.79 respectively. The number of inflammatory cells increased significantly between the OA1 and OA3 groups (*p* < 0.05) (Fig. 1).

### Metabolomic analysis

**Figure 2:**
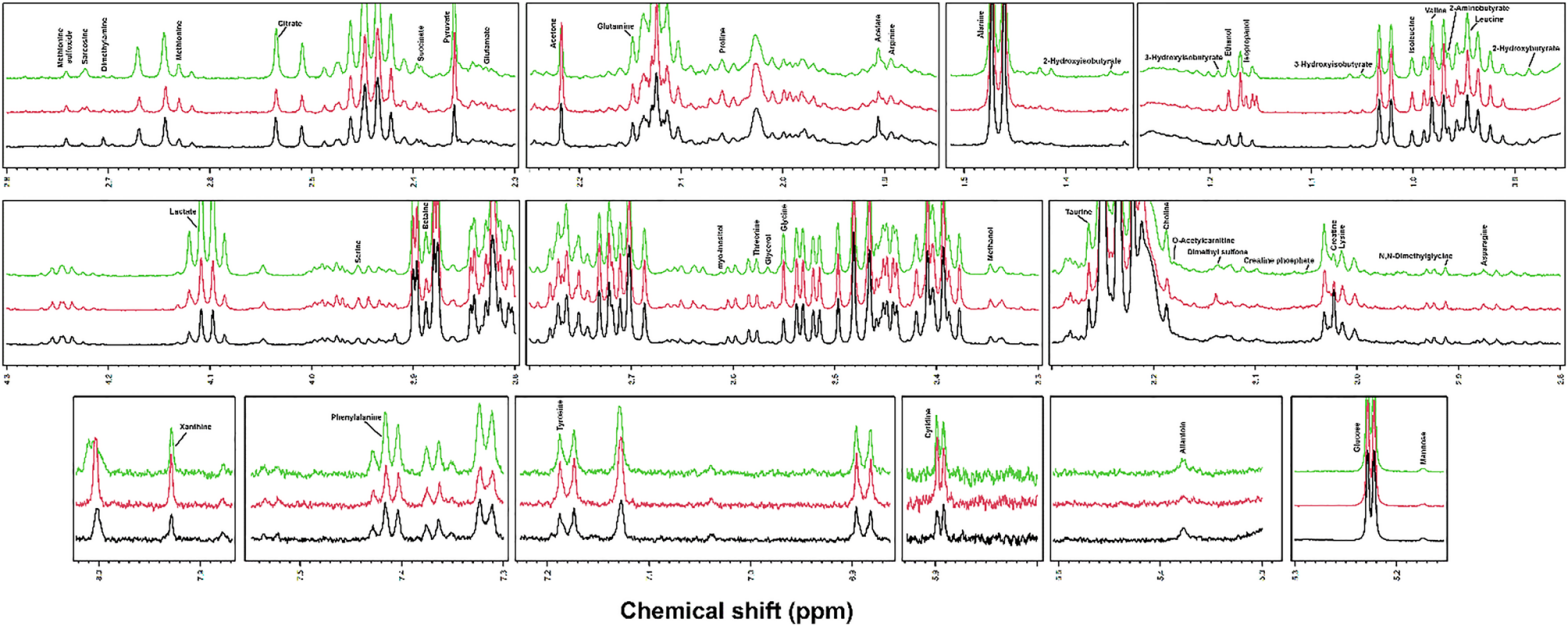
Representative ^1^H-NMR spectra of OA1 (black), OA2 (red), and OA3 (green) SF samples.

Forty-eight metabolites were identified and quantified in the SF sample during ^1^H-NMR metabolomic analysis (Fig. 2).

**Figure 3:**
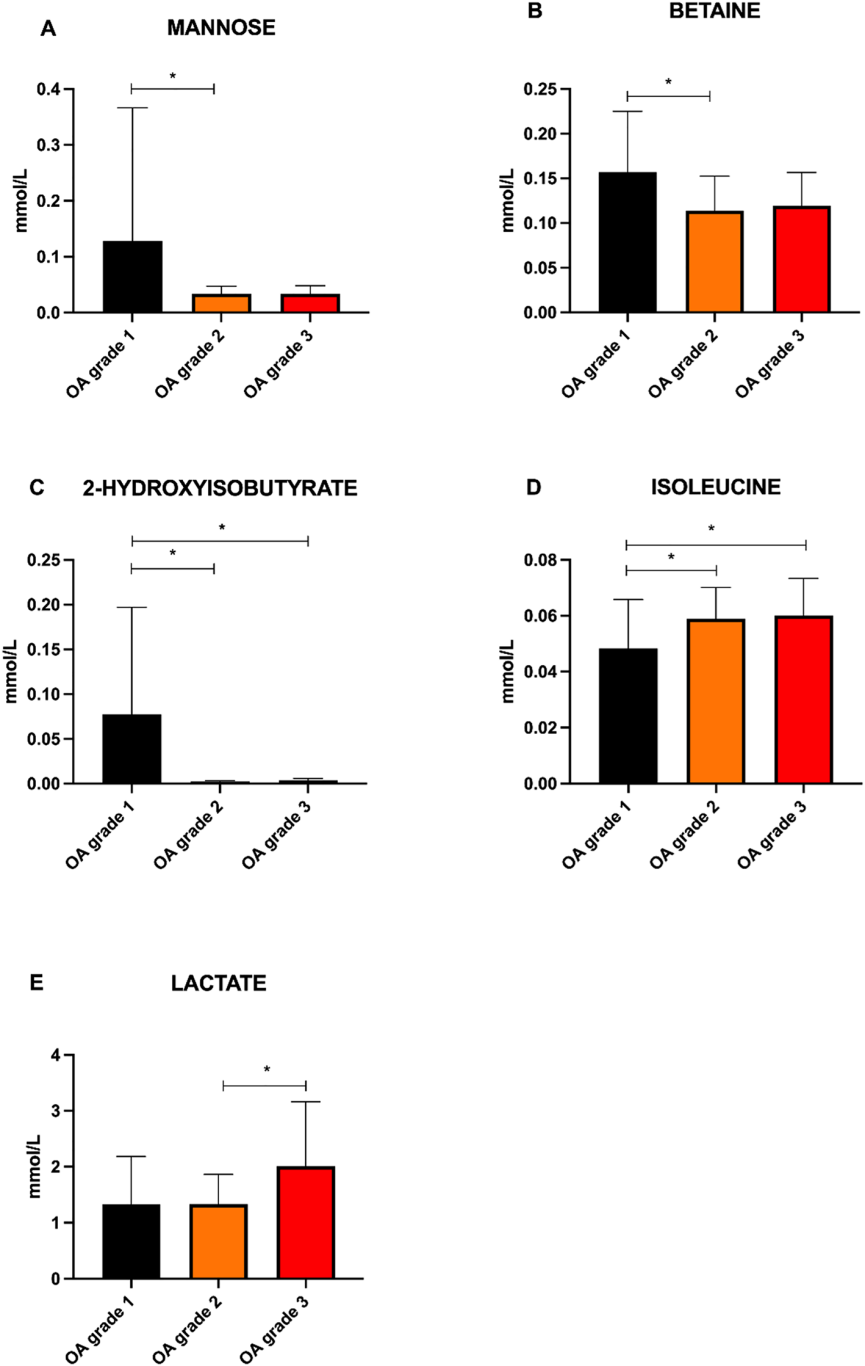
Comparison of the concentrations (mmol/L) of mannose (**A**), betaine (**B**), 2-hydroxyisobutyrate (**C**), isoleucine (**D**), and lactate (**E**) (means and SEM) between OA1(n = 14), OA2 (n = 29), and OA3 (n = 17) groups. Asterisk (*) indicates a significant difference (*p* < 0.05) between groups.

The concentrations of mannose, betaine, 2-hydroxyisobutyrate, isoleucine, and lactate were significantly different between the OA1, OA2, and OA3 groups (*p* < 0.05) (Table 2, Fig. 3). Between the OA1_stifle_, OA2_stifle_ and OA3_stifle_ groups, the concentration of mannose, betaine, 2-hydroxyisobutyrate, lactate, o-acetylcarnitine and pyruvate were significantly different (*p* < 0.05) (Table 3).

**Table 2:** Concentration of metabolites (mean ± SD mmol/L) identified during ^1^H-NMR metabolomic analysis of canine SF samples.

Metabolite	OA1 group (mmol/L)	OA2 group (mmol/L)	OA3 group (mmol/L)	ANOVA *p* value	Chemical shift (ppm)	Level of identification
2-Aminobutyrate	0.023 ± 0.016	0.026 ± 0.013	0.026 ± 0.015	0.740	0.9653	2
2-Hydroxybutyrate	0.024 ± 0.023	0.021 ± 0.015	0.025 ± 0.013	0.703	0.8855	2
2-Hydroxyisobutyrate	**0.087 ± 0.129**^**a,b**^	**0.002 ± 0.001**^**a**^	**0.004 ± 0.002**^**b**^	**0.011**	**1.3553**	**2**
3-Hydroxybutyrate	0.016 ± 0.016	0.020 ± 0.025	0.018 ± 0.009	0.765	1.1906	2
3-Hydroxyisobutyrate	0.017 ± 0.022	0.012 ± 0.005	0.014 ± 0.005	0.397	1.0496	2
Acetate	0.025 ± 0.008	0.031 ± 0.013	0.027 ± 0.007	0.239	1.9070	1
Acetone	0.014 ± 0.006	0.017 ± 0.013	0.017 ± 0.011	0.716	2.2188	1
Alanine	0.380 ± 0.163	0.409 ± 0.114	0.455 ± 0.102	0.232	1.4728	1
Allantoin	0.055 ± 0.040	0.040 ± 0.024	0.045 ± 0.044	0.940	5.3773	1
Arginine	0.188 ± 0.201	0.156 ± 0.109	0.110 ± 0.049	0.229	1.8935	1
Asparagine	0.053 ± 0.022	0.045 ± 0.027	0.042 ± 0.020	0.457	2.8739	1
Betaine	**0.157 ± 0.068**^**a**^	**0.114 ± 0.039**^**a**^	**0.119 ± 0.037**	**0.020**	**3.8880**	**1**
Cytidine	0.040 ± 0.015	0.024 ± 0.010	0.031 ± 0.020	0.129	3.1871	2
Citrate	0.113 ± 0.035	0.183 ± 0.310	0.116 ± 0.023	0.483	2.5372	1
Choline	0.016 ± 0.018	0.011 ± 0.008	0.013 ± 0.012	0.518	3.0228	1
Creatine	0.036 ± 0.026	0.049 ± 0.057	0.030 ± 0.016	0.325	3.0326	1
Creatine phosphate	0.044 ± 0.017	0.047 ± 0.023	0.048 ± 0.014	0.853	5.8960	1
Dimethyl sulfone	0.017 ± 0.032	0.014 ± 0.040	0.008 ± 0.003	0.678	3.1391	1
Dimethylamine	0.002 ± 0.002	0.002 ± 0.001	0.002 ± 0.001	0.363	2.7036	1
Ethanol	0.140 ± 0.166	0.235 ± 0.355	0.314 ± 0.355	0.335	1.1820	1
Glycerol	0.183 ± 0.150	0.184 ± 0.136	0.280 ± 0.330	0.289	5.2215	1
Glycine	0.208 ± 0.084	0.192 ± 0.065	0.213 ± 0.075	0.604	2.3277	1
Glucose	3.003 ± 1.305	3.006 ± 1.042	2.726 ± 0.634	0.635	2.1475	1
Glutamate	0.249 ± 0.620	0.086 ± 0.046	0.078 ± 0.048	0.199	3.5655	1
Glutamine	0.493 ± 0.150	0.498 ± 0.098	0.484 ± 0.146	0.993	3.5499	1
Isoleucine	**0.048 ± 0.017**^**ab**^	**0.059 ± 0.011**^**a**^	**0.060 ± 0.013**^**b**^	**0.031**	**1.0017**	**1**
Isopropanol	0.020 ± 0.011	0.017 ± 0.013	0.019 ± 0.016	0.935	1.1647	1
Lactate	**1.333 ± 0.852**	**1.33 ± 0.516**^**c**^	**2.040 ± 1.144**^**c**^	**0.015**	**4.1094**	**1**
Leucine	0.106 ± 0.034	0.119 ± 0.021	0.123 ± 0.033	0.242	0.9464	1
Lysine	0.078 ± 0.031	0.090 ± 0.021	0.076 ± 0.018	0.196	3.0150	1
*N*,*N*-Dimethylglycine	0.004 ± 0.003	0.003 ± 0.002	0.004 ± 0.001	0.452	5.1736	1
Methionine	0.040 ± 0.018	0.037 ± 0.010	0.047 ± 0.015	0.095	3.3467	1
Methionine sulfoxide	0.019 ± 0.013	0.016 ± 0.011	0.022 ± 0.026	0.571	2.6306	1
Mannose	**0.128 ± 0.239**^**a**^	**0.034 ± 0.013**^**a**^	**0.034 ± 0.014**	**0.034**	**2.7414**	**1**
Methanol	0.073 ± 0.106	0.040 ± 0.012	0.052 ± 0,020	0.150	3.6114	1
Myo-inositol	0.118 ± 0.231	0.060 ± 0.035	0.075 ± 0.053	0.290	2.9126	1
O-Acetylcarnitine	0.005 ± 0.003	0.005 ± 0.010	0.004 ± 0.002	0.860	3.1788	2
Phenylalanine	0.052 ± 0.015	0.056 ± 0.013	0.055 ± 0.013	0.766	7.4167	1
Pyruvate	0.055 ± 0.041	0.045 ± 0.027	0.062 ± 0.015	0.182	2.0588	1
Proline	0.136 ± 0.033	0.120 ± 0.033	0.138 ± 0.027	0.071	2.3597	1
Sarcosine	0.014 ± 0.015	0.010 ± 0,013	0.011 ± 0.008	0.704	2.7241	1
Serine	0.208 ± 0.072	0.214 ± 0.060	0.214 ± 0.067	0.943	3.9527	1
Succinate	0.010 ± 0.006	0.014 ± 0.018	0.009 ± 0.006	0.367	2.3922	1
Taurine	0.166 ± 0.073	0.152 ± 0.070	0.156 ± 0.070	0.824	3.2636	1
Threonine	0.291 ± 0.086	0.303 ± 0.206	0.264 ± 0.100	0.729	3.5761	1
Tyrosine	0.040 ± 0.015	0.048 ± 0.020	0.042 ± 0.020	0.424	7.1876	1
Valine	0.137 ± 0.053	0.164 ± 0.031	0.166 ± 0.032	0.059	0.9822	1
Xanthine	0.027 ± 0.019	0.023 ± 0.013	0.029 ± 0.010	0.303	7.9254	1

The superscript letters indicate a significant difference (*p* < 0.05) between OA1(n = 14), OA2 (n = 29), and OA3 (n = 17) groups, in particular the letter “a” (^a^) indicates a significant difference between OA1 and OA2 groups, the letter “b” (^b^) indicates a significant difference between OA1 and OA3 groups, and the letter “c” (^c^) indicates a significant difference between OA2 and OA3 groups. In the sixth column is reported the chemical shift of resonances that were used for quantification and in the last column is reported the level of identification of the metabolites as set out Metabolomics Standards Initiative (MSI). Significant values are in bold.

**Table 3:** Concentration of metabolites (mean ± SD mmol/L) significant difference (*p* < 0.05) between OA1_stifle_ (n = 11), OA2 _stifle_ (n = 21), and OA3 _stifle_ (n = 13) groups, identified during ^1^H-NMR metabolomic analysis of stifle joint SF samples.

Metabolite	OA1_stifle_ group (mmol/L)	OA2_stifle_ group (mmol/L)	OA3_stifle_ group (mmol/L)	ANOVA* p*-value
2-Hydroxyisobutyrate	0.102 ± 0.169^a,b^	0.002 ± 0.001^a^	0.004 ± 0.003 ^b^	0.046
Betaine	0.155 ± 0.077^a,b^	0.100 ± 0.035^a,c^	0.115 ± 0.034^b,c^	0.013
Lactate	1.038 ± 0.543^a,b^	1.141 ± 0.422^a,c^	1.675 ± 0.686^b,c^	0.009
Mannose	0.151 ± 0.267^a,b^	0.036 ± 0.014^a^	0.038 ± 0.014^b^	0.052
O-Acetylcarnitine	0.006 ± 0.003^a.b^	0.003 ± 0.001^a,c^	0.004 ± 0.002^b,c^	0.008
Pyruvate	0.045 ± 0.033^a.b^	0.036 ± 0.017^a,c^	0.060 ± 0.014^b,c^	0.013

The superscript letter “a” (^a^) indicates a significant difference between OA1_stifle_ and OA2_stifle_ groups, the superscript letter “b” (^b^) indicates a significant difference between OA1_stifle_ and OA3 _stifle_ groups, and the letter superscript “c” (^c^) indicates a significant difference between OA2_stifle_ and OA3_stifle_ groups.

The concentration of mannose in canine SF was significantly elevated (*p* = 0.034) in the OA1 group (0.128 ± 0.239 mmol/L) compared with OA2 (0.034 ± 0.013 mmol/L). In the OA3 group, the mannose concentration was lower (0.034 ± 0.014 mmol/L), but not significantly, compared with the OA1 group (Fig. 3A). The same evolution could be observed for the concentration of mannose in the SF of stifle joints, because it was significantly higher (*p* = 0.052) in the OA1_stifle_ group (0.151 ± 0.267 mmol/L) than in the OA2_stifle_ group (0.036 ± 0.014 mmol/L) and in the OA3_stifle_ group (0.038 ± 0.014 mmol/L).

As the mannose, the concentration of metabolite betaine showed a significantly higher concentration (*p* = 0.0196) in the OA1 group (0.157 ± 0.068 mmol/L) than in the OA2 group (0.112 ± 0.038 mmol/L), but there was no statistically significant difference between the OA1 and OA3 groups (0.123 ± 0.038 mmol/L) (Fig. 3B), unlike what was observed in the SF of the stifles, where the difference was also significant between OA1_stifle_ (0.115 ± 0.077 mmol/L) and OA3_stifle_ groups (0.115 ± 0.034 mmol/L).

The OA progression led to a significant decrease in the concentration of 2-hydroxyisobutyrate: a significantly higher concentration could be observed in the OA1 group (*p* = 0.011; 0.087 ± 0.129 mmol/L) than in the OA2 (0.002 ± 0.001 mmol/L) and the OA3 groups (0.004 ± 0.002 mmol/L) (Fig. 3C), and in the OA1_stifle_ group (*p* = 0.046; 0.102 ± 0.169 mmol/L) than in the OA2_stifle_ group (0.002 ± 0.001 mmol/L) and the OA3_stifle_ group (0.004 ± 0.003 mmol/L).

An opposite evolution is observed for concentrations of isoleucine and lactate. Isoleucine significantly increased (*p* = 0.0313) with the OA progression. In particular, its concentration was significantly lower in the OA1 group (0.048 ± 0.017 mmol/L) compared to the OA2 group (0.05952 ± 0.011 mmol/L) and in the OA1 group compared to the OA3 grous (0.059 ± 0.013 mmol/L) (Fig. 3D). In the OA3 group, lactate showed a significantly higher concentration (2.008 ± 1.118 mmol/L) compared with the OA2 group (1.335 ± 0.525 mmol/L) (*p* = 0.0186) (Fig. 3E). Different was the significance observed in the concentration of these two metabolites in the SF of the only stifle joints. Although isoleucine tends to increase with the progress of OA in the stifles, this increase is not statistically significant (OA1_stifle_: 0.050 ± 0.019 mmol/L; OA2_stifle_: 0.058 ± 0.011 mmol/L; OA3_stifle_: 0.062 ± 0.014 mmol/L), while the concentration of lactate revealed a significant increment between all study groups (*p* = 0.009; OA1_stifle_: 1.038 ± 0.543 mmol/L; OA2_stifle_: 1.141 ± 0.422 mmol/L; OA3_stifle_: 1.675 ± 0.686 mmol/L).

From the comparison between the concentrations of the metabolite o-acetylcarnitine and pyruvate in the SF of the stifle joints, a significant initial decrease in these metabolites was observed (o-acetylcarnitine: OA1_stifle_ 0.006 ± 0.003 mmol/L, OA2_stifle_ 0.003 ± 0.001 mmol/L; pyruvate: OA1_stifle_ 0.045 ± 0.033 mmol/L; OA2_stifle_ 0.036 ± 0.017 mmol/L), and then a subsequent significant increase was detected with OA progression (o-acetylcarnitine: OA3_stifle_ 0.004 ± 0.002 mmol/L; pyruvate: OA3_stifle_ 0.060 ± 0.014 mmol/L).

## Discussion

This study demonstrated biochemical differences in the ^1^H-NMR metabolomic profile of canine SF in patients with progressive degrees of spontaneous OA. To our knowledge, this is the first study in domestic animals that quantified and compared different metabolite concentrations in progressive OA stages, and, in particular, the first study that compared three stages of osteoarthritic joints. In humans, Kim et al.^31^ investigated changes in metabolite concentrations in SF in progressive stages of human knee OA using the Kallgren-Lawrence scale, identifying 28 metabolites, related to fatty acid metabolism, glycerolipid metabolism and tricarboxylic acid cycle, as important molecules for discriminating between the early and late OA stages. In our study, 48 metabolites were identified in SF samples, and five of them showed a significant shift during OA progression: mannose, betaine, 2-hydroxyisobutyrate, isoleucine, and lactate. Furthermore, pyruvate and o-acetylcarnitine exhibited significant shifts during OA progression only in the SF of the evaluated stifle joints.

Mannose is a simple sugar, five times more active than glucose in non-enzymatic glycation^32^, and is the major monosaccharide component of the N-glycans^33^. The high-mannose type N-glycans are more highly localized in the superficial chondrocytes^34^. The OA changes, which were characterized by a decrease in the extracellular matrix, chondrocyte apoptosis, and dedifferentiation, were accompanied by a decrease in high-mannose type N-glycans in chondrocytes throughout the entire cartilage layer^35^. Urita et al.^35^ observed that the high-mannose type N-glycan structure is altered during the processes of human articular cartilage degradation, and that the related N-glycogen (GlcNAc-TI), an enzyme that converts high-mannose type N-glycans to complex-type N-glycan, influences the regulation of MMP-13 and ADAMTS-5 (aggrecanase 2) expression in mouse chondrocytes in response to stimulation with IL-1α. These findings suggest that high-mannose type N-glycans are key molecules in the initiation and pathogenesis of OA^35^, and the sudden decrease of concentration of mannose observed in our study in advanced stages of OA could be explained by increased demand for mannose used for N-glycosylation in response to the alteration of N-glycans localized in superficial chondrocytes.

Furthermore, mannose is phosphorylated by hexokinase (HK) to produce mannose-6-phosphate (Man-6-P) directed into N-glycosylation via phosphomannomutase (PMM2), but it can also be catabolized by phosphomannose isomerase (MPI) within the cell^33^. Saito et al.^36^ profiled sugar metabolism in leukemia cells and found that mannose is an energy source for glycolysis thanks to leukemia cells’ high levels of expression of phosphomannose isomerase (PMI). With regard to these recent studies, the use of mannose as an energy source for glycolysis could be a possible explanation for its reduced concentration in SF, but to be able to affirm these hypotheses, further studies are needed to assess the mechanism of N-glycosylation and mannose consumption and to assess the activity of MPI in the intraarticular environment during OA.

Different metabolomic studies have also observed the increment of isoleucine with the OA progression. In particular, increased levels of branched-chain amino acids (BCAAs), such as isoleucine, leucine, and valine, in OA patients could be due to either blocked catabolism of BCAAs or increased release from the breakdown of proteins^15^. The BCAAs are essential amino acids making up one-third of skeletal muscle protein and are relevant for energy metabolism^15^. An animal model study of OA showed an enhancement of the resonance at 0.85 ppm of the _1_H high-resolution magic angle spinning NMR spectra of the OA-affected cartilage sample, which could be attributable to the increase in leucine and isoleucine^54^, suggesting the association in the current study could be due to the release of amino acids from joint collagen breakdown. Studies of osteoarthritic canine SF also showed an increased concentration of isoleucine, supporting this hypothesis^24^. Recently, the ratio of BCAAs to the amino acid histidine was suggested as a biomarker for OA: the increase in serum BCAAs correlated with OA’s radiographic severity^22^. BCAAs have been shown to increase the production of cytokines, including interleukin 1 (IL-1) and 2 (IL-2), TNF-α, and IFN-γ^40^. It could be possible that an increased concentration of BCAAs leads to increased production of cytokines, which leads to an increased rate of joint collagen degradation associated with OA^22^. Our results showed a significant increment of isoleucine and a slight increment of valine and leucine, supporting a possible alteration of BCAA metabolism in the OA progression.

In previous studies, the concentration of ketone bodies increased in osteoarthritic SFs compared to healthy SFs, suggesting that fat metabolism plays an important role as a source of energy in OA joints^7^. In particular, hydroxybutyrate and other ketone bodies are a part of the regulatory mechanism affecting glucose and lipid metabolism. The ketone bodies decrease glucose utilization and pyruvate oxidation, maintaining normal glycolytic intermediates for biosynthetic purposes^24^. The results of our study demonstrated a significant decrease in the concentration of 2-hydroxyisobutyrate: a significantly higher concentration can be observed in the OA1 group compared with more advanced stages of pathology (OA2 and OA3 groups). 2-hydroxyisobutyrate is a molecule that mediates lysine 2-hydroxyisobutyrylation, one of the newly identified post-translational modifications (PTMs), which are important epigenetic modifications of the nucleosomal core histones^44^. The study of histone modification has emerged as a new field in the context of OA^46^. Some epigenetic changes are considered possible causes of the abnormal gene expression and subsequent alteration of the chondrocyte phenotype (hypertrophy, proliferation, senescence) and extracellular matrix homeostasis, as observed in osteoarthritic cartilage^46^. In the intraarticular environment, the PTMs, such as lysine 2-hydroxyisobutyrylation, activate fibroblastic proliferation and migration, reduce cartilage erosion by decreasing the production of interferon γ (IFN-γ) in natural SF killer cells, and reduce the formation of osteoclasts, which mediate bone erosion in rheumatoid arthritis models^47^. It could reasonably be assumed that 2-hydroxyisobutyrate has a protective role. In addition, in the mouse model of OA, hydroxyisobutyrylation also reduces the production of TNF-α in macrophages, increasing the synthesis of collagen (COL2A1 and COL10A1) and glycosaminoglycan in chondrogenic mesenchymal stem cells, protecting from cartilage erosion. Hydroxyisobutyrylation finally inhibits the expression of matrix metallopeptidases 9 and 13 (MMP-9, MMP-13) and a disintegrin and metalloproteinase with type 1 motif 5 (ADAMTS-5) in joint chondrocytes^46^. Therefore, a progressive reduction of 2-hydroxyisobutyrate during the OA progression, as suggested by our data, could promote the activation of all factors favoring inflammation and articular cartilaginous dystrophy, reducing the activation of the arthro-chondroprotection factors. Using 2-hydroxyisobutyrate for the lysine 2-hydroxyisobutyrylation could explain this decrease in its concentration.

Betaine, a stable and nontoxic natural substance widely distributed in animals, plants, and microorganisms, is an important methyl group donor in transmethylation, a process catalyzed by betaine-homocysteine methyltransferase (BHMT), and it is an essential osmoprotectant, primarily in the kidneys, liver, and brain. A large amount of betaine can accumulate in cells without disrupting cell function and protects cells under osmotic stress^50^. In particular, it can increase cells’ cytoplasmic volume and free water content to prevent shrinkage in hyperosmotic conditions^51^. Bush et al*.* observed that the volume of chondrocytes within the superficial and mid-zones increased with cartilage degeneration. Cell swelling was greater than that expected from the increased hydration in OA, suggesting that increased chondrocyte volume might arise from factors other than increased cartilage hydration, such as elevated cell osmolyte concentration^53^. Betaine could participate in this mechanism and accumulate within chondrocytes, justifying its decreased concentration in the SF of the OA2 group, but not its mild increment in the OA3 group. In addition, it could also be justified because betaine could be used for its anti-inflammatory effect. It inhibits the nuclear factor-κB (NF-κB) activity, which controls many genes involved in inflammation: the pro-inflammatory cytokines tumor necrosis factor-α (TNF-α), interleukin 1β (IL-1β), interleukin 23 (IL-23), and leucine-rich pyrin-containing 3 (NLRP3) inflammasome activation^54^. In contrast to our study, a recent metabolomic article reported a lack of significance between betaine concentration in healthy and osteoarthritic joints, but reported a lower concentration of choline in the OA joints^27^. The betaine content of body is heavily influenced by the amount of betaine consumed through diet^54^, but it can be also synthesized from choline in the mitochondria^54^, so its concentrations could influence betaine. Our results suggested a decrease of betaine concentration in the OA2 group with respect to the OA1 groups, and the same evolution can be observed for the concentration of the metabolite choline, but without significantly different concentrations between study groups.

In addition, the concentration trend of betaine and choline showed an important analogy with the evolution of concentration of methionine in the SF of dogs with OA, attributed to their interconnection within the *methionine synthesis cycle*. The methionine is involved in complex metabolic pathways^56^, including the production of the methyl donor S-adenosylmethionine (SAME), which is the source of the three methyl groups of choline by transmethylation^57^. Choline is a fundamental donor of methyl (CH_3_) groups after oxidation to betaine for the re-methylation of homocysteine to methionine. In practice, in the presence of choline or betaine, the body could methylate the sulfur of homocysteine to give methionine^58^. Many studies indirectly corroborate the decrease in choline found in our study because betaine is derived from choline, and this latter metabolite is also positively related to lipid metabolism and inflammation^59^. Other studies corroborate our observations also regarding the dramatic decrease of hydroxybutyrate metabolites and the decrease of choline (fundamental for phosphatidylcholine synthesis), as well as betaine and methionine^61^.

Our investigations focused on potential correlations between metabolite alterations and the degree of articular involvement in canine progressive OA, reflecting the severity of inflammation and immune cells within the inflamed joints. Normally, during OA1 to OA3 progression, different leukocytes are recruited to the site of inflammation, including macrophages, CD4+ and CD8+ T cells, B cells, and neutrophils^63^. Resident macrophages, the predominant immune cell type in chronic joint diseases, and mast cells, proliferate during chronic inflammation while neutrophils are still present, but their percentage is lower than that in the acute state due to the presence of other recruited cell types^66^. In our study, the chronic higher degree of canine joint inflammation enhances macrophage recruitment, as observed by cytology in fact an increasing number of inflammatory cells were in observed in OA2 and OA3 groups compared to OA1 group. In vitro studies have demonstrated that methionine sulfoxide treatment alters the extracellular nucleotide metabolism, promoting an increase in ATPase/ADPase activities in macrophages, promoting alterations in the redox state of macrophages, and increasing reactive oxygen and nitrogen species (RONS) levels^62^, and it is known that M1/classical macrophage activation is related to pro-inflammatory responses characterized by increased iNOS activity and TNF-α release^64^. Our data showed a not statistically significant increase of concentration of methione sulfoxide, but we could observe that different levels of chronic inflammation led to differences in the immune cell composition, illustrated by the changes in the concentration of metabolites involved in redox, immune, energy, cell growth, and nucleotide metabolism.

Currently, doubts are being raised about the role of lactate as a discriminating factor for an osteoarthritic joint. Lactate represents the end product of glycolysis, and anaerobic conditions favor its production. Different authors have documented increased concentrations of lactate in the SF of an osteoarthritic joint and a reduced concentration of glucose, suggesting an intraarticular environment more hypoxic and acidotic than that of a healthy joint^24^. In contrast, some authors have not found such differences in lactate concentration^25^. Considering the inconsistent results in the literature and our significant increase in lactate concentration, especially in the severe stage of of OA, it is likely that lactate is a discriminating factor for the most advanced stages of OA, rather than for mild pathological conditions. In fact, lactate accumulation has been observed during chronic inflammation^69^. This metabolite, associated with an accumulation of pyruvate in OA3 group, significantly noticeable in the stifle joints, indicates a progressive disruption of the tricarboxylic acid (TCA) cycle, reflecting a response to increased energy consumption and reduced fatty acid reserves. The high increase in lactate concentration in SF during the progression of OA could also be explained by conversion of pyruvate to lactate due to the inflammatory peak and the necessary stabilization of the acidic environment to recruit additional inflammatory cells^24^.

The OA is characterized by alterations in energy metabolism and cloud contribute to changes in o-acetylcarnitine levels. In the SF of the stifle joints, affected by rupture of the cranial cruciate ligament, o-acetyl-carnitine decreases during the OA progression, only to show a slight increase in subjects with severe OA. The o-acetylcarnitine (or acetyl-L-carnitine) is a specific form of acylcarnitine that is derived from the acylation of carnitine with acetic acid^71^. It is involved in cellular energy metabolism, in particular transports the acyl groups from the cytosol into the mitochondrial matrix for β-oxidation and it is a regulator of energy metabolism controlling the Acyl-CoA:CoA ratio^37^. Inflammatory and degenerative processes in the joint may require increased use or consumption of o-acetylcarnitine to respond to tissue damage and energy demand. As a result, o-acetylcarnitine levels in SFs may decrease. On the contrary, the slight increase that we observed in the more advanced stages of OA, can be explained by an alteration of mitochondrial proteins identified in advanced OA in a recent proteomic study of mitochondria from normal or osteoarthritic chondrocytes^37^. Further research is needed to better clarify the role of o-acetylcarnitine in pathogenesis and progression of joint disease.

Some studies suggest a potential beneficial effect of supplementation or restriction of some of these metabolites on OA^47^. Fontana et al.^73^ suggested that restriction of BCAA intake might provide novel nutraceutical approaches to OA management. Recently, Zhou et al*.*^74^ demonstrated that exogenous administration of D-mannose in a mouse model alleviates OA progression by suppressing the hypoxia-inducible factor 2α (HIF-2α) mediated chondrocyte sensitivity to ferroptosis. Ferroptosis is a form of oxidative cell death characterized by the iron-dependent accumulation of lipid hydroperoxides to lethal levels^74^, which contributes to the progression of OA^75^. The decrease in the SF concentration of mannose during the progression of OA observed in our data and the capacity of exogenous administration of D-mannose to alleviate OA progression, thanks to its therapeutic strategy for ferroptosis-related diseases, could make the mannose metabolic pathway an interesting therapeutic target to investigate^74^. Besides betaine’s anti-inflammatory, antifibrotic, and antiangiogenic properties, Yajun et al*.* demonstrated its capacity to suppress osteoclastogenesis in vitro by inhibiting reactive oxygen species (ROS) production and subsequent mitogen-activated protein kinase (MAPK) signaling, in addition to having anti-inflammatory, antifibrotic, and antiangiogenic properties^76^. These data demonstrated that betaine attenuated OA progression by inhibiting hyperactivated osteoclastogenesis and maintaining microarchitecture in the subchondral bone. This study on the exogenous administration of betaine in a mouse model with OA^76^ and the decreased betaine concentration in our results may further prompt us to consider this metabolite a potential therapeutic target. Future analysis of altered metabolic pathways during OA progression may highlight their potential therapeutic effect^15^.

Stifle joints, affected by rupture of cranial cruciate ligament, represented the majority of the joints included in our study. The cranial cruciate ligament disease is cause of pelvic limb lameness; it is associated with joint instability and it leads to secondary pathologic change such as OA^80^. The results of the statistical comparison of the concentration of metabolites identified in the SF of the stifles were similar to the results obtained from the comparison of all joints, however, the significant difference was found for the pyruvate and o-acetylcarnitine concentrations, where the significant difference was not detected from the first statistical comparison of all included joints. So, localization or pathology could affect the metabolome, but further studies are needed to assess how the localization or the rupture of the cruciate ligament may affect the metabolome, in particular studies that compare healthy stifles with affected stifles. The same analysis should be performed for other joints affected by other conditions.

Our study has some limitations. First, the metabolomic profiles of dogs of different breeds that were enrolled could be different, as previously demonstrated^18^; secondly, we have not analyzed the potential influence of body condition score on the metabolome of the SF^18^. In addition, the clinical nature of the study and the standards recommended by EU Directive 2010/63/EU for experiments on animals did not allow us to have a control group to include healthy dogs’ joints without OA. The execution of an arthrocentesis of a healthy joint, without clinical or radiographic indication, does not comply with animal welfare laws, and the difficulty of obtaining the SF of dogs subjected to euthanasia free of metabolic pathologies is evident. It is not yet clear the mechanism of action of these metabolites in relation to the evolution of OA, so some considerations are hypothetical and should therefore be contextualized.

In conclusion, this study emphasized the presence of different ^1^H-NMR metabolomic profiles of canine SF in patients with progressive degrees of spontaneous OA. The results suggested the existence of therapeutic potentials of the metabolic pathways that involve mannose, betaine, choline, 2-hydroxyisobutyrate, isoleucine, and lactate, and monitoring their concentrations can give information on the evolution of OA.

In both humans and domestic animals, this is the first study to quantify and compare different metabolite concentrations in three stages of osteoarthritic joints, encouraging the application of metabolomic analysis in clinician research for diagnosis, monitoring of progression, and treatment of OA. Further studies are needed to assess the actual therapeutic potential of the involved metabolic pathways.

## Materials and methods

### Ethics statement

This study was performed in accordance with relevant guidelines and regulation and it was approved by the Institutional Animal Care and Use Committee of the University of Camerino (protocol no. 11/2023) according to the standards recommended by EU Directive 2010/63/EU for experiments on animals.

### Animals

Client-owned dogs showing lameness in one limb and affected by spontaneous OA were referred to the Veterinary Teaching Hospital of the University of Camerino and prospectively enrolled in the study. The inclusion criteria were dogs aged 1 to 15 years, with no weight and sex restriction, belonging to the ASA 1–2 anesthetic risk class^82^. The presence of comorbidity, pregnancy, or lactation; the presence of OA in other joints of the same limb; and joints affected by arthropathies not on a degenerative basis, such as septic arthritis, immunomediated arthritis, hemarthrosis, and neoplasia, represented exclusion criteria. Dogs that had received anti-inflammatory drugs or nutraceuticals in the 20 days before enrollment or intraarticular treatments in the 180 days before enrollment were excluded from the study.

### Clinical, radiographic, and synovial cytologic examinations

Clinical, radiographic, and cytologic evaluations were performed to confirm the OA diagnosis.

Anamnestic and signaling data were collected. Subsequently, an expert orthopedic surgeon performed clinical and radiographic examinations under anesthesia (more than 20 years of experience).

Patients were sedated with 2 μg/kg of dexmedetomidine and 0.2 mg/kg of methadone IM and then anesthetized by 2–3 mg/kg of propofol IV until tracheal intubation was achieved. Anesthesia was maintained with 1.2% isoflurane in oxygen for the radiographic evaluation. The radiographic examination was performed, and orthogonal projections of the affected joint were obtained from each patient to blindly assess the OA according to the modified Kellgren–Lawrence scale (Table 4): score 0 = normal (grade 0); score 1–3 = mild OA (grade 1); score 4–6 = moderate OA (grade 2); score 7–9 = severe OA (grade 3); score > 10 = extremely severe/deforming OA (grade 4)^13^.

**Table 4:** Modified Kellgren–Lawrence scale.

Radiographic sign	0	1	2	3	4
Osteophytes	Absent	< 1 mm	1–2 mm	2–3 mm	> 3 mm
Bone sclerosis	Absent	Localized	Pervasive		
Joint narrowing and/or incongruence	Absent	Mild < 25%	Moderate 25–50%	Serious > 50%	Joint deformity
Capsular ectasia	Absent	Evident	–	–	–
Final score	0	1–3	4–6	7–9	> 10
OA grade	0	1	2	3	4

**Figure 4:**
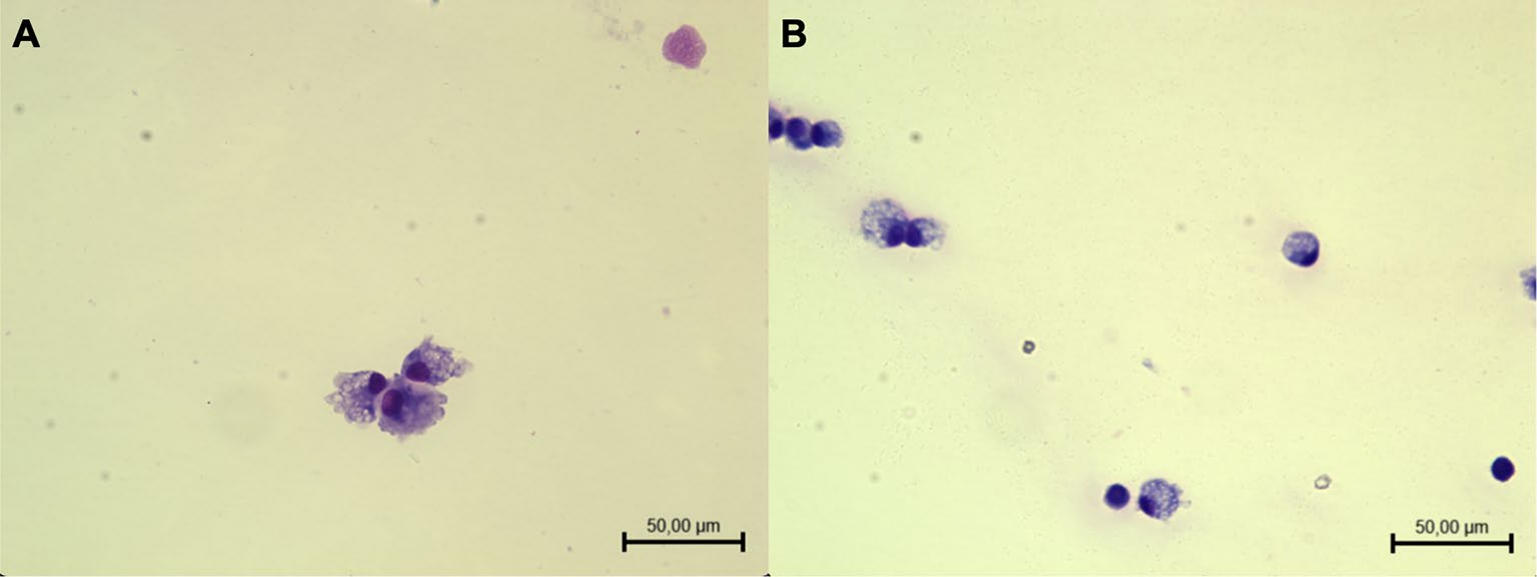
Two synovial fluids with degenerative arthropathy characterized by several macrophages with abundant vacuolized cytoplasm and prominent pseudopods (**A**) and rare mature lymphocytes (**B**) on a background with poor protein material (May-Grümwald Giemsa staining).

During anesthesia, the affected joint was trichotomized and SF samples were collected aseptically by arthrocentesis for qualitative and semiquantitative cytological examination and metabolomic analysis. An aliquot of SF was blindly assessed in the Veterinary Pathology Unit of the University of Camerino to characterize the type of disease affecting the joint (for diagnostic purposes), and subsequently, joints affected by arthropathies different from OA, such as septic or immunomediated arthropathy, hemarthrosis, and neoplasia, were excluded from the study. Only joints with cytologic findings of degenerative arthropathy characterized by an increased number of nucleated cells with a predominance of vacuolated macrophages (Fig. 4) were included in the study. For each sample the number of mononuclear cells (macrophages and lymphocytes) was counted in 5 representative high-power filed (40X) and reported as the mean ± SD. A second aliquot was collected into 0.4 mL Eppendorf tubes and stored at − 80 °C until metabolomics analysis via ^1^H-NMR was performed.

**Figure 5:**
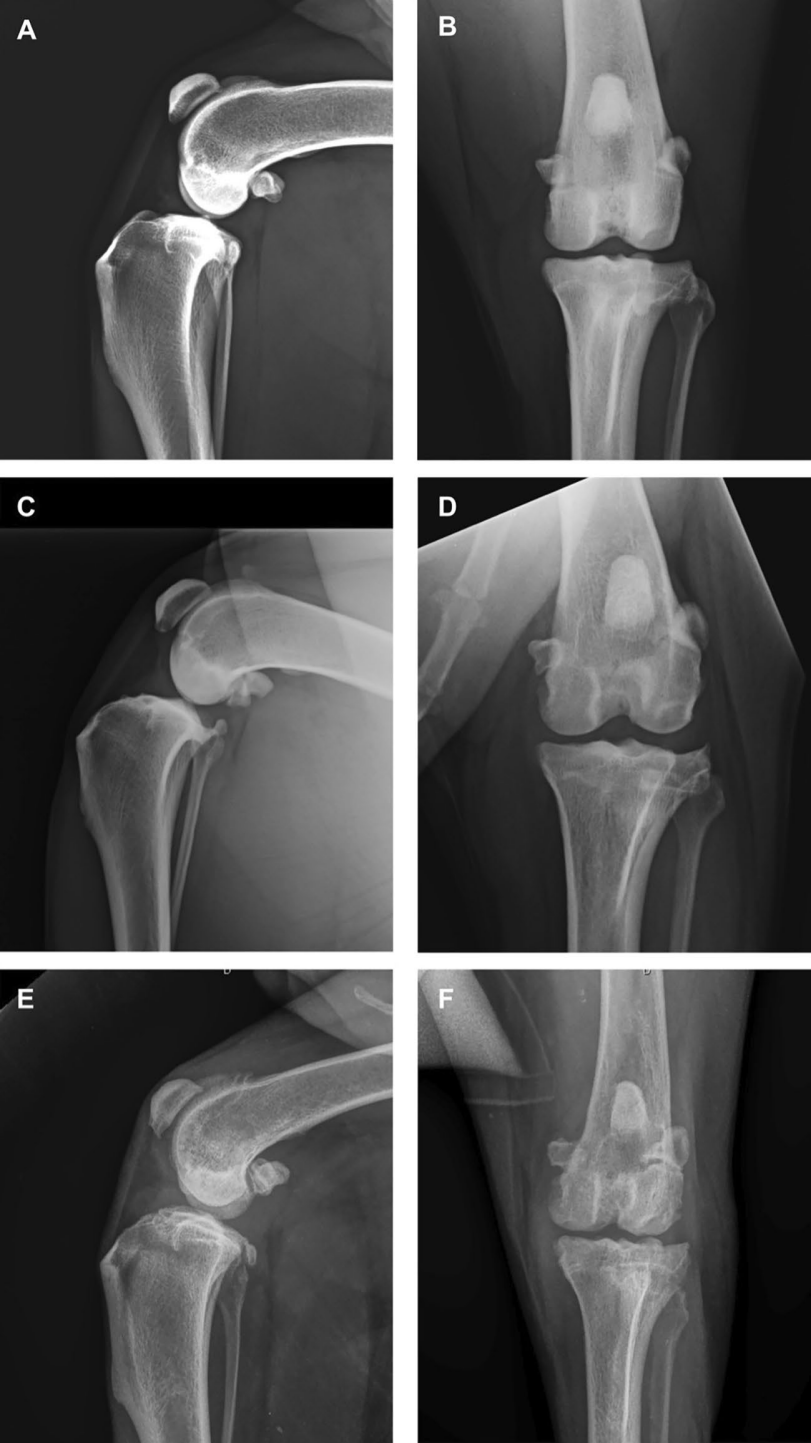
Medio-lateral (ML) (**A**) and caudo-cranial (CaCr) (**B**) radiographic projections of a stifle joint with mild OA according to the modified Kellgren–Lawrence scale (OA1 group); ML (**C**) and CaCr (**D**) radiographic projections of a knee joint with moderate OA according to the modified Kellgren–Lawrence scale (OA2 group); ML (**E**) and CaCr (**F**) radiographic projections of a stifle joint with severe OA according to the modified Kellgren–Lawrence scale (OA3 group).

When the OA diagnosis was confirmed, the recruited affected joints were definitively divided into four study groups of OA grade (according to the modified Kellgren–Lawrence scale): OA1 group (mild OA), OA2 group (moderate OA), OA3 group (severe OA), and OA4 group (extremely severe/deforming OA) (Fig. 5).

### Synovial metabolomic analysis

For the observation of the metabolome of synovial fluids, an NMR analysis solution with 3-(trimethylsilyl)-propionic acid-2,2,3,3-d_4_ sodium salt (TSP) 10 mM in D_2_O was prepared and then set at pH 7 with a 1 M phosphate buffer. The solution contained 0.01 mL of 2 mM NaN_3_ to avoid microbial proliferation. SF samples were prepared for ^1^H-NMR analysis by centrifuging 0.4 SF with 0.4 mL of H_2_O (2540×*g*; 300 s; 4 °C). The obtained solution was centrifuged (18,630×*g*; 900 s; 4 °C), and 0.6 mL of supernatant was added to 0.1 mL of ^1^H-NMR analysis solution. As described above, each sample was centrifuged once again.^84^

^1^H-NMR spectra were registered (600.13 MHz; 298 K) with an AVANCE™ III spectrometer (Bruker, Milan, Italy) set at a frequency of 600.13 MHz and operated using Topspin v3.5 software^84^. A CPMG filter of 330 ms allowed the suppression of signals with a very short relaxation time due to large molecules. In addition, a presaturation allowed suppression of the residual water signal. The double suppression was achieved by the cpmgpr1d sequence, part of the standard pulse sequence library. Each spectrum was acquired by summing up 256 transients constituted by 32,000 data points over a 7184 Hz spectral window, and then phased^84^.

Assignment of signals to specific molecules was performed in Chenomx software (Chenomx Inc., Canada, ver. 8.3) by comparison with the internal database (ver. 10) and the HMDB database^85^ implemented in Chenomx (version 2), together with previous investigations on the same biofluid of horses^27^. The assignment strategy, utilizing multiple sources, enabled us to categorize the rigor of each assignment as either level 1 or 2, in accordance with the scale introduced by Sumner et al.^86^ After bringing the spectra into the R environment^87^ through custom scripts, quantification was performed in one sample with a median water dilution, assessed through probabilistic quotient normalization (PQN)^88^, by relying on the TSP area and known concentration. The area of each signal was measured by global spectra deconvolution, implemented in the MestReNova software (Ricerca Mestrelab S. L. Santiago De Compostela (Spain), ver. 14.2.0-26256). This was done after applying a line-broadening noise reduction of 0.3 and a baseline adjustment through the Whittaker smoother procedure.

### Statistical analysis

Data about the presence and concentration of metabolites detected in SF samples were analyzed using the GraphPad Prism 9 software (GraphPad Software, Inc., La Jolla, CA, USA). All data are presented as mean ± standard deviation (SD) and were first checked for normality using the Kolmogorov–Smirnov test. An Ordinary one-way ANOVA followed by the Tukey multiple comparison test was used to compare differences in metabolite concentration among the groups with different degrees of OA (OA1, OA2 and OA3 groups) and among the groups with different degrees of OA considering only the stifle joints (OA1_stifle_, OA2 _stifle_ and OA3 _stifle_ groups). Conversely, a Kruskal–Wallis test followed by the Dunn’s multiple comparison test was used to compare differences in synovial mononuclear cells among the groups the three aforementioned groups. The level of significance was set at *p* < 0.05.

### Institutional review board statement

This study was approved by the Institutional Animal Care and Use Committee (OPBA) of the University of Camerino (Protocol No. 11/2023) according to the standards recommended by EU Directive 2010/63/EU for experiments on animals. This study is reported in accordance with ARRIVE guidelines.

## Data Availability

The data present in this study are available within the article.
